# Diagnosis and Treatment of Pancreas Rejection

**DOI:** 10.1007/s40472-015-0061-x

**Published:** 2015-04-11

**Authors:** R. R. Redfield, D. B. Kaufman, J. S. Odorico

**Affiliations:** Division of Transplantation, Department of Surgery, University of Wisconsin–Madison School of Medicine and Public Health, University of Wisconsin Hospital and Clinics, 600 Highland Ave, Clinical Science Cntr-H4/756, Madison, WI 53792 USA

**Keywords:** Diagnosis, Treatment, Pancreas rejection

## Abstract

Despite significant improvement in pancreas allograft survival, rejection of the pancreas remains a major clinical problem. In addition to cellular rejection of the pancreas, antibody-mediated rejection of the pancreas is now a well-described entity. The 2011 Banff update established comprehensive guidelines for the diagnosis of acute and chronic AMR. The pancreas biopsy is critical in order to accurately diagnose and treat pancreas rejection. Other modes of monitoring pancreas rejection we feel are neither sensitive nor specific enough. In this review, we examine recent advances in the diagnosis and treatment of pancreas rejection as well as describe practical diagnostic and treatment algorithms.

## Introduction

Solid organ pancreas transplant outcomes continue to improve [1], with the half-life of simultaneous pancreas and kidney (SPK) allograft approaching 15 years and that of solitary pancreas recipients nearing 13 years [2]. Much of this success can likely be attributed to improved surgical technique, immunosuppression, and diagnosis and treatment of rejection. In this review, we will specifically focus on recent advances in the diagnosis and treatment of pancreas rejection.

## Clinical Presentation of Rejection

Clinically, pancreas allograft function is commonly monitored by a number of parameters including hyperglycemia, serum amylase, serum lipase, C-peptide level, hemoglobin A1C, or, if bladder drained, urinary amylase. However, in order to accurately identify and define rejection, these clinical tools are insufficient, because they are too non-specific. There are multiple potential causes for hyperamylasemia, hyperlipasemia, and hyperglycemia. Even stable kidney function, or a normal serum creatinine in SPK transplants, is not a consistently reliable indicator that pancreatic graft immunological damage is not occurring [4]. Indeed, it is not uncommon to observe isolated pancreas rejection in the setting of normal kidney allograft function (Table 1).

**Table 1:** Pancreas allograft biopsies at the University of Wisconsin from January 1994 to July 2012

Total	422
Percutaneous	406 (96.2 %)
Rejection (of any kind)	265 (62.8 %)
With normal amylase and lipase	20 (7.5 %)
Pancreatitis	16 (3.8 %)
Non-diagnostic	24 (5.7 %)
Simultaneous pancreas and kidney biopsies	20
Discordant for rejection	5 (25 %)

**Fig. 1:**
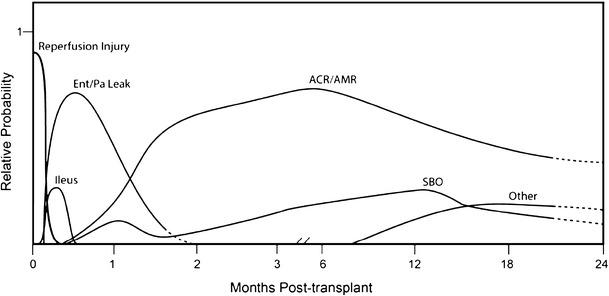
Temporal relationship of elevated pancreas enzymes to etiology. Surgical complications typically present early in the postoperative course, whereas rejection most commonly presents later. “Other” includes possible causes such as transplant pancreatic duct stricture, native pancreatitis, IPMN or cancer in pancreas transplant or native pancreas, and penetrating ulcer. Of note, although complete graft thrombosis can present as increased pancreatic enzymes, in our experience, this is a very uncommon presentation, and when most grafts thrombose, very limited increases in enzymes are seen if at all. Relative probability on the *Y*-axis does not represent the overall probability or incidence of this complication; instead, it strives to convey the relative probability that these diagnoses are associated with elevated pancreatic enzymes. Abbreviations: *ACR/AMR* acute cellular rejection/antibody-mediated rejection, *Ent/Pa Leak* enteric or pancreatic leak, *SBO* small bowel obstruction

Elevated pancreatic enzymes is the most common presentation of pancreatic allograft rejection, and most patients are asymptomatic or have only mild graft tenderness upon presentation. If a patient is asymptomatic, the source of the elevated enzymes is more likely to be from the transplanted pancreas as the graft itself is insensate. In contrast, symptomatic increases in pancreatic enzymes can point to either transplant or native gland disease. Lipase is considered to be more specific than amylase and has greater sensitivity in the author’s experience. Naturally, these signs and symptoms can arise from multiple causes in the setting of an enterically drained graft. Hence, it is useful to consider the differential diagnosis and the timing of presentation when evaluating these patients (Fig. 1, Table 2).

**Table 2:** Common diagnoses contributing to increased pancreatic enzymes after pancreas transplantation

Diagnosis	Overall frequency (%)	Frequency the entity presents as increased enzymes
Perioperative (<45 days)		
Enzyme leak^1^	∼5	High
Infected fluid/abscess	∼5	Moderate
Thrombosis	2–5	Low
Ileus	5–10, transient	Moderate
Acute rejection	1–2	High
Mid postoperative (>45 days–1 year)		
Acute rejection^a^	15–20	High
SBO	5	Low–moderate
Pseudocyst	2–5	High
Constipation	2–5	Low–moderate
Abscess	2–5	Low–moderate
CMV pancreatitis	∼1	High
Late postoperative (>1 year)		
Acute rejection	5–10	High
Chronic rejection	∼5	Moderate–high
SBO/ventral hernia	∼10	Low–moderate
Intrinsic pancreatic abnormality^b^	∼5	Moderate–high
Native pancreatitis	1	High
CMV pancreatitis	<1	High

^1^Enzyme leak—enteric or parenchymal, including pseudocyst or pancreatic ascites

^a^Frequency depends on risk factors such as type of transplant, primary vs. re-transplant, sensitized status, donor-specific antibody, race mismatch, and donor age

^b^Includes graft trauma, chemical pancreatitis, pancreatic duct stricture, IPMN, carcinoma, pseudocyst

Abbreviations: *SBO* small bowel obstruction, *CMV* cytomegalovirus

**Fig. 2:**
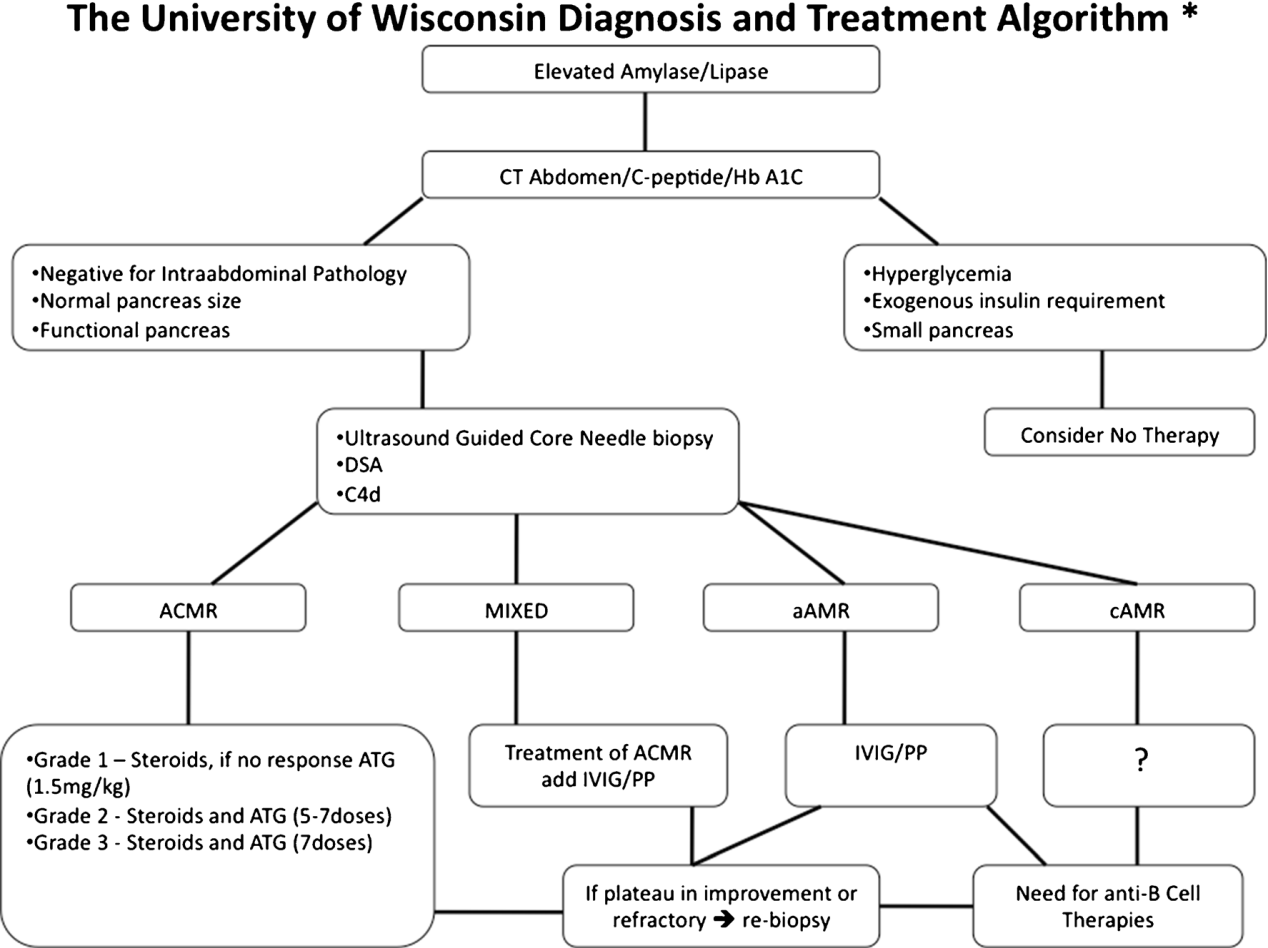
The University of Wisconsin Diagnosis and Treatment Algorithm Abbreviations: *DSA* donor-specific antibody, *ACMR* acute cell-mediated rejection, *aAMR* acute antibody-mediated rejection, *cAMR* chronic antibody-mediated rejection, *ATG* anti-thymocyte globulin, *IVIg* intravenous immunoglobulin, *PP* plasmapheresis. [(Published in Trends in Transplantation, © Permanyer Publications) 2].

An algorithm for the diagnosis and management of patients with increased pancreatic enzymes is shown in Fig. 2 [3•]. In our practice, the initial approach to the patient with elevated enzymes is history and physical, fasting C-peptide, HbA1C, donor-specific antibodies (DSA), and an imaging study, preferably CT scan of the abdomen and pelvis with IV and PO contrast (Fig. 2). In the perioperative period (<45 days) or in the presence of abdominal symptoms, it is important to obtain a CT scan first before allograft biopsy to evaluate for postsurgical complications or intra-abdominal infection. On the other hand, if the patient is beyond the perioperative period and without abdominal symptoms, a surgical cause for the elevated enzymes is significantly less likely, and therefore, it is reasonable to go straight to pancreas allograft biopsy and assess the graft for anatomical abnormalities during performance of the biopsy. Regardless of the time frame posttransplant, a pancreas allograft biopsy and CT scan will efficiently identify the cause for elevated pancreatic enzymes in the vast majority of cases.

Medication non-compliance, recent intolerance of oral medications, alcohol consumption, recent abdominal trauma, constipation, presence of a ventral hernia, abdominal pain, nausea, vomiting, and obstructive symptoms are important points to elicit from the patient. Duodenal or parenchymal enzyme leak, pseudocyst, mycotic aneurysm, SBO, intrinsic pancreatic abnormalities such as duct strictures, and tumors are included in the differential diagnosis of elevated enzymes and are usually diagnosed with imaging studies.

In our experience, transplant pancreatitis that is not attributable to an anatomic or immunological cause is very uncommon. Instead, transplant pancreatitis, putatively defined as increased pancreatic enzymes without evidence of other anatomic abnormality on CT scan other than peri-pancreatic transplant inflammation and graft swelling, can also be indicative of acute or chronic rejection or CMV pancreatitis. In our large series of for-cause pancreas allograft biopsies, non-immunological pancreatitis was only diagnosed on biopsy in 4 % (16/422) of biopsies, a frequency similar to that reported previously [4]. Therefore, transplant pancreatitis should be considered a diagnosis of exclusion (Table 1).

While most patients who have acute rejection present with elevated pancreatic enzymes, it is not uncommon to see patients develop chronic rejection and graft failure without significant increases in their serum enzymes. Why chronic rejection does not always coincide with serological evidence of acinar injury is not presently known, but may be related to progressive loss of acinar/parenchymal tissue resulting in less release of enzymes and/or reduced lab frequency resulting in missed detection.

## Pancreas Allograft Biopsy

In order to diagnose pancreas rejection accurately, a biopsy of the pancreas allograft is necessary. It is currently the only available means of grading the severity of rejection and distinguishing antibody-mediated rejection (AMR) from acute cellular rejection (ACR). The most commonly performed approach to allograft biopsy is percutaneous ultrasound-guided biopsy of the pancreas parenchyma, which can be performed in either bladder-drained or enterically drained allografts safely and effectively [5, 6]. Alternative methods of acquiring tissue from the pancreaticoduodenal allograft have been described. These include percutaneous CT-guided biopsy, endoscopic biopsy of the donor duodenum for enterically drained pancreata, or, if bladder drained, cystoscopic needle biopsy of pancreatic head and/or mucosal biopsy of the donor duodenum. The potential for discordant findings between pancreas histology and duodenal histology [7–9] has limited the widespread reliance of sampling duodenal mucosa for diagnosis. Furthermore, C4d staining features characteristic of AMR in the duodenal mucosa have not been adequately defined, and current methods for staining duodenal mucosal biopsies for C4d may not be sufficiently specific. Additionally, using the kidney as a sentinel organ, as can be done in SPK transplants, can be misleading. In our experience, ∼25 % of kidney and pancreas biopsies in SPK patients are discordant (Table 1). Prior studies have demonstrated that isolated pancreas rejection with normal renal function in SPK patients is not uncommon [4, 10]. Therefore, we advocate for an actual core biopsy of pancreatic tissue to rule out rejection.

In our practice, we prefer ultrasound-guided biopsy with an 18-gauge automatic biopsy device. Our trajectory for biopsy is ideally towards the tail, avoiding the splenic artery and vein. Because of the nest of vessels and larger duct in the head region, we prefer to biopsy the body/tail of the graft aiming either transversely or preferably longitudinally with respect to the axis of the pancreas. We have performed 406 percutaneous biopsies since 1992, with a very low complication rate and no graft losses. Two patients required reoperation: one for bleeding and one for evacuation of pancreatic ascites, which ultimately resolved, and both patients currently still retain excellent graft function. If we are unsuccessful, or there is no suitable safe window free of overlying bowel, then we proceed with CT-guided biopsy using a posterior approach, or alternatively an open or laparoscopic biopsy, but this is required rarely. The majority of pancreas biopsies were diagnostic, only 6 % (24/422) were non-diagnostic (Table 1).

## Distinguishing Histologic Features of AMR, ACR, and Pancreatitis

The 2007 Banff guidelines for the diagnosis of pancreas rejection solidified prior histopathological descriptions and focused on ACR [11–13]. However, in response to reports in the literature documenting pancreas AMR, the 2011 Banff update established comprehensive guidelines for diagnosis of acute and chronic AMR [14] of the pancreas (Table 3). While a complete review of current histopathological criteria of rejection is beyond the scope of this review, suffice it to say that the only definitive way to precisely diagnosis the severity and mechanism of pancreas allograft rejection is with a pancreas biopsy. In our series of 422 for-cause biopsies, 63 % were diagnostic for rejection; only 4 % were determined to be graft pancreatitis (Table 1).

**Table 3:** Banff 2011 classification of cell- and antibody-mediated rejection of the pancreas

1. Acute T cell-mediated rejection
Grade I/mild acute T cell-mediated rejection—active septal inflammation (activated, blastic lymphocytes, and ±eosinophils) involving septal structures: venulitis (subendothelial accumulation of inflammatory cells and endothelial damage in septal veins, ductitis (epithelial inflammation and damage of ducts) *and*/*or* Focal acinar inflammation. No more than two inflammatory foci per lobule with absent or minimal acinar cell injury Grade II/moderate acute T cell-mediated rejection (requires differentiation from AMR) Multifocal (but not confluent or diffuse) acinar inflammation (≥3 foci per lobule) with spotty (individual) acinar cell injury and dropout *and*/*or* Mild intimal arteritis (with minimal, <25 % luminal compromise) Grade III/severe acute T cell-mediated rejection (requires differentiation from AMR) Diffuse (widespread, extensive) acinar inflammation with focal or diffuse multicellular/confluent acinar cell necrosis *and*/*or* moderate or severe intimal arteritis, >25 % luminal compromise *and*/*or* Transmural inflammation—necrotizing arteritis
2. Antibody-mediated rejection
(a) Confirmed circulating donor-specific antibody (DSA)
(b) Morphological evidence of tissue injury (interacinar inflammation/capillaritis, acinar cell damage, swelling/necrosis/apoptosis/dropout, vasculitis, thrombosis)
(c) C4d positivity in interacinar capillaries (IAC, ≥5 % of acinar lobular surface)
Acute AMR 3 of 3 diagnostic components Consistent with acute AMR 2 of 3 diagnostic components Requires exclusion of AMR 1 of 3 diagnostic components See below for histological grading of acute AMR Chronic active antibody-mediated rejection: combined features of categories 1 and 2 in the absence of features of ACMR
3. Chronic allograft rejection/graft fibrosis
Stage I (mild graft fibrosis) Expansion of fibrous septa; the fibrosis occupies less than 30 % of the core surface but the acinar lobules have eroded, irregular contours. The central lobular areas are normal Stage II (moderate graft fibrosis) The fibrosis occupies 30–60 % of the core surface. The exocrine atrophy affects the majority of the lobules in their periphery (irregular contours) and in their central areas (thin fibrous strands crisscross between individual acini) Stage III (severe graft fibrosis) The fibrotic areas predominate and occupy more than 60 % of the core surface with only isolated areas of residual acinar tissue and/or islets present
Histological grading of acute AMR
Grade I/mild acute AMR
Well-preserved architecture, mild monocytic-macrophagic or mixed (monocytic-macrophagic/neutrophilic) infiltrates with rare acinar cell damage
Grade II/moderate acute AMR
Overall preservation of the architecture with interacinar monocytic-macrophagic or mixed (monocytic-macrophagic/neutrophilic) infiltrates, capillary dilatation, capillaritis, congestion, multicellular acinar cell dropout, and extravasation of red blood cells
Grade III/severe acute AMR
Architectural disarray, scattered inflammatory infiltrates in a background of interstitial hemorrhage, multifocal and confluent parenchymal necrosis, arterial and venous wall necrosis, and thrombosis

(With permission from: Drachenberg CB, Torrealba JR, Nankivell BJ, Rangel EB, Bajema IM, Kim DU, et al. Guidelines for the diagnosis of antibody-mediated rejection in pancreas allografts—updated Banff grading schema. American Journal of Transplantation. 2011;11 (9):1792–802)]]

## Incidence and Risk Factors for Rejection

Niederhaus et al. recently reported on the frequency of both cellular and antibody-mediated pancreas allograft rejection as they relate to specific risk factors [15••]. The incidence of rejection within 1-year posttransplant in a cohort of 162 patients of all pancreas transplant types including many re-transplants was 21 %, with antibody-mediated rejection (AMR), acute cellular rejection (ACR), and mixed rejection occurring in nearly equal frequency. In their study, the majority of pancreas rejection episodes were successfully reversed and graft function was maintained; however, 20 % of grafts were lost within a year of diagnosis.

Dong et al. similarly showed a 1-year acute rejection rate of 14.7 % increasing to 26.6 % by 5 years posttransplant [16]. The findings of Dong et al. also support the notion that allograft rejection even if diagnosed and treated aggressively is associated with pancreas graft failure in a subset of patients especially if diagnosed beyond 3 months posttransplant [11].

Risk factors for rejection identified in these two studies include non-primary SPK transplants, primary solitary pancreas alone (PTA), race mismatch [10], and increasing donor age [15••, 16]. Increased vigilance for rejection in these scenarios, which are associated with higher immunological risk, may therefore be warranted.

## Donor-Specific Antibody as a Biomarker of Rejection

The deleterious effects of both pre-formed donor-specific antibodies (DSA) and de novo DSA are well established in the kidney transplant literature [17]. Few studies, however, have directly evaluated the role of DSA in pancreas transplantation. Cantarovich et al. showed that DSA in SPK transplants was an independent predictor of graft failure. Of their patients, 24 % (40/167, 152 were SPK recipients) were found to have DSA postoperatively. Maintenance therapy was with anti-thymocyte globulin, tacrolimus, and mycophenolate mofetil only. This study identified DSA as an independent predictor of graft failure [18]. However, DSA was not quantified in all patients preoperatively so it is unclear from this study what proportion of recipients developed de novo DSA vs. those who already had pre-formed DSA.

Mittal et al. demonstrated in a large cohort of pancreas transplant patients that de novo DSA was also an independent risk factor for graft loss [19•]. In this study of 439 pancreas transplant patients (73 % SPK), de novo DSA developed in 37.9 % of patients of the patients who had follow-up. The DSA MFI cutoff was 1000. Pancreas biopsies in association with elevated DSA were not reported in this study. The immunosuppressive regimen consisted of alemtuzumab induction followed by MMF and tacrolimus maintenance immunosuppression. Steroids were not used as maintenance therapy. This is a surprisingly high rate of de novo DSA, and one wonders if it was causally associated with this alemtuzumab/steroid-free immunosuppressive regimen. There are other reports of alemtuzumab associated with increased DSA production in kidney transplant recipients, which may explain such an elevated incidence of de novo DSA [20]. Obviously, this area requires further study to understand the exact mechanisms of these observations, especially the association and timing of de novo DSA in relation to histopathological rejection. But nonetheless, strategies to prevent or reduce de novo DSA may lead to better graft outcomes.

In our practice, the presence of pretransplant DSA aids our assessment of the posttransplant immunologic risk. For patients with pretransplant DSA and a negative flow crossmatch, we tend to favor using a depleting antibody for induction therapy. We monitor all our patients with DSA measurements postoperatively with the frequency of monitoring determined by a preoperative risk stratification. However, it is currently unknown what to do with de novo DSA in the setting of normal pancreas allograft function. One hypothesis is that it indicates under immunosuppression, and increasing immunosuppression maybe warranted. Elevated DSA in the setting of normal graft function could also be a harbinger of eventual graft dysfunction or simply could be of no clinical consequence. Certainly more studies are needed. If the emergence or rise in DSA does in fact accompany abnormal graft function, then a biopsy is warranted to rule out rejection.

## Treatment of Pancreas Rejection

A normal fasting C-peptide and HbA1c confirms a functioning pancreas allograft prior to the initiation of anti-rejection therapy. Consideration of pancreas graft functional reserve prior to initiating any anti-rejection therapy is paramount. Hyperglycemia, as evidenced by significantly elevated fasting blood glucose or HbA1c, or low fasting C-peptide level should be taken into account when weighing the potential benefits and risks of pursuing therapy. Additionally, a significant chronic component to the patient’s rejection status, as evidenced by fibrotic changes on biopsy, should factor into the potential benefit of therapy. Treatment of chronic or acute rejection if significant hyperglycemia is present prior to initiation of steroid therapy, or if the patient is on insulin, is controversial, but traditionally, hyperglycemia had been thought to be a marker of poor salvagability in a pancreas allograft [12]. If the patient’s graft dysfunction has progressed to the point of requiring large doses of exogenous insulin, forgoing treatment for rejection should be considered, especially since several therapeutic agents will only exacerbate hyperglycemia. If the graft has evidence of significant fibrosis, and/or a paucity of islets and/or is small on imaging, and C-peptide is low, then chronic rejection is probable and little will be gained with treatment, or until at least better therapies are developed. On the other hand, if the patient has only minimal fibrosis and primarily acute changes, with detectable normal appearing islets, imaging that shows a normal-sized gland, and fasting C-peptide is easily detectable well into the normal range, then it is worth treating the rejection process aggressively in our experience even with the short-term prospect of worsening glycemic control and temporary insulin use.

H&E staining can diagnose and grade ACR, and we treat accordingly with steroids +/− anti-thymocyte globulin (ATG) as illustrated in Fig. 2. It is important to note that, if ACR is present, it should be treated aggressively. In the setting of mixed cellular and AMR, we recommend that initial therapy be directed against the cellular component. Niederhaus et al. found, even in cases of mixed rejection, the pancreas graft survival mirrored that of ACR alone if treated [15••]. The incidence of mixed rejection is not trivial, occurring in 7 % of recipients at 1 year. At this time, it is unclear if mixed rejection requires IVIG, plasmapheresis, and/or specific anti-B cell therapies or if steroids and ATG is sufficient. If the biopsy is negative or indeterminate for ACR, we await results of C4d immunostaining and DSA, which usually takes a day or two longer in our laboratory.

If DSA is positive with histologic findings consistent with AMR, even in the absence of C4d, we proceed with treatment consisting of IVIG and plasmapheresis as we would in the setting of significant C4d staining. In cases of a mixed rejection, we generally add ATG. If the amylase/lipase decline, we continue treatment until they have normalized. If their descent stalls or if they start to rise again, we usually repeat the biopsy. If the re-biopsy demonstrates persistent AMR, additional anti-B cell therapies, such as bortezomib, rituximab, or eculizumab, are considered. Admittedly, treatment of pancreas AMR is currently pursued without clear data and the aforementioned approach is merely proposed as a guide. Even in the kidney AMR literature, there is a paucity of randomized clinical trails. Despite this, there have been advances in the treatment of AMR resulting in improved outcomes. The main treatment goal in AMR is for the reduction of DSA and elimination of the B cell and plasma cell population responsible for the production of DSA. Hopefully, carefully protocolized treatments and the examination of outcomes will be prospectively conducted.

Overall, key points of our treatment algorithm (Fig. 2) are as follows: ensure the pancreas is still functioning and therefore worthy of salvage, obtain tissue diagnosis to evaluate the degree of ACR and AMR, and treat ACR aggressively as a first and primary strategy. Sequentially, escalate therapy as data becomes available and/or if there is no improvement in enzymes with ACR-directed treatment. Finally, if there is any doubt as to the cause of ongoing enzyme elevation or hyperglycemia, it is valuable to consider re-biopsy. While we acknowledge this field is still evolving, we believe the aforementioned approach is a reasonable management protocol (Fig. 2).

## Conclusions

In our practice of evaluating each patient for the possibility of pancreas graft rejection, we gather serologic and histologic data in the form of DSA and biopsy, and if hyperglycemic, then fasting C-peptide and HbA1c. Once a diagnosis of rejection is made, we treat accordingly as described in Fig. 2 for ACR, AMR, and mixed types of rejections, as long as the pancreas is worth saving (i.e., no evidence of hyperglycemia or elevated HbA1c). One strategy is to use currently available anti-T and anti-B cell-directed therapies sequentially, progressively escalating therapies over the course of several days to a week, depending on the response and relative contribution of each component to the active rejection process. However, it should also be kept in mind that clear data is lacking in this area. Moving forward, more rigorous study is required to define the best treatment regimens for rejection.
